# Applying a general systems theory framework in mental health treatment pathways: the case of the Hellenic Center of Mental Health and Research

**DOI:** 10.1186/s13033-020-00398-z

**Published:** 2020-08-17

**Authors:** Panagiotis Katrakazas, Aliki Grigoriadou, Dimitrios Koutsouris

**Affiliations:** 1 Biomedical Engineering Laboratory, Athens, Greece; 2 Hellenic Center for Mental Health and Research, Athens, Greece

**Keywords:** Mental health, Operational framework, General systems theory, Systems approach, Children population

## Abstract

Language, socio-emotional and cognitive development in children and adolescents with mental health issues is getting increased attention over the last years. Establishing communication patterns and addressing behavioural diversities among this population should be of priority, along with a better understanding in a large variety of patient characteristics within the operational framework of mental healthcare centers*.* Therefore, the relationships between provided services and operational capability should become more evident. As integrated systems’ approaches are still missing to predict the efficiency of treatment services in a macroscopic scale, a General Systems Theory framework is hereby proposed. This framework is applied and tested against the operational framework of the Hellenic Center of Mental Health and Research, in order to identify the need of such an approach and the strong cooperation between medical and population interactions. Using such frameworks as a prerequisite to identify important factors affecting population states can lead to evaluating their impact on the treatment outcome and depict the complexity of pathways potentially related to the children’s development.

## Highlights

- Sensitive groups of children population require a multidisciplinary approach
- Operational frameworks in mental health could improve through a systemic approach
- A general systems theory framework is proposed and applied to a Greek mental health research center
- Mental health care and research can benefit from a state and time-perspective data basis

## Background

According to World Health Organization (WHO),^1^ childhood and adolescence are considered to be critical stages for the development of skills in self-control, social interaction and learning. This can affect the mental health and well-being of children and adolescents, whereas exposure to risk factors (e.g. bullying at school) can negatively affect them in the long-term. As the rates of mental health and behavioural problems at the population level are high and continue to increase, healthcare systems could benefit from employing additional tools and methodologies.

Two of the objectives identified in the WHO’s comprehensive mental health plan [1] refer to (a) the provision of comprehensive and integrated mental health and social care services and (b) the strengthening of evidence-based information systems. In order to analyse and assess the functionality and operational capability of mental healthcare services, identification of correlations and relationships within such systems would provide a better insight related to the services provided. Such an approach would then easily provide an evidence-base for the consequences of a high socioeconomic burden of mental diseases, apart from the medical and emotional one, not only on the micro- (e.g. individuals and their families) level, but on the meso- (e.g. school population) and macro- (e.g. nation) level as well.

It is the aim of the current paper to suggest an integrated system’s approach based on General Systems Theory to show that the application and exploitation of such frameworks in a very specialized and focused attempt can help to define the individual and population relationships, characteristics and interactions inside and outside a system. Moreover, the assessment of the efficiency and operational capability within an existing mental healthcare provision center allows the observation and identification of a correspondence between social, economic and operational indicators. A description of these parameters are provided hereinafter, based on semi-structured interviews with the staff of the Hellenic Center of Mental Health and Research and online material provided by their website (https://www.ekepsye.gr/).

### Description of the operational framework within the Hellenic Center of Mental Health and Research (HCMHR)

**Fig. 1:**
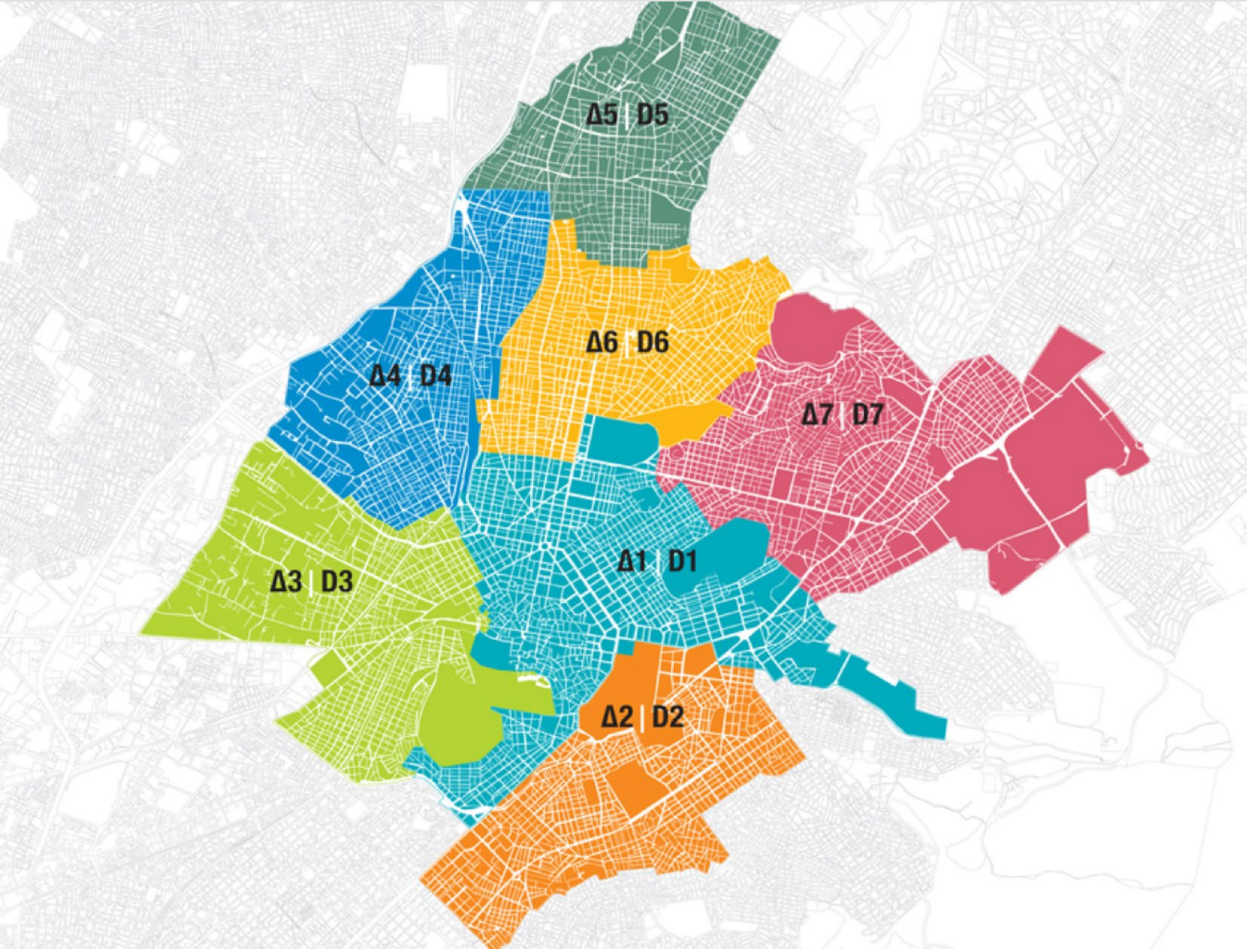
Municipal Communities of Athens, Greece (https://modmov.ellet.gr/maps/)

The Hellenic Center of Mental Health and Research (HCMHR) is a Mental Health Unit of the broader Public Sector and it is under the jurisdiction and financial support of the Greek Ministry of Health, which has eight Mental Health Units in total nationwide. The specific unit is located in the center of Athens and serves three of the seven municipal communities of the Municipality of Athens (Fig. 1). More specifically it serves the fourth, fifth and sixth municipal districts, with the following characteristics:

1. 4th Municipal Community (D4): It includes the western districts (Kolonos, Platonos Academy, Kolokynthos, Prophet Daniel, Sepolia, Nirvana). Its population according to the 2011 census is 85,629 (compared to 2001: 92,310).
2. 5th Municipal Community (D5): It includes the northwestern districts from Kato Patisia to Probona (Agios Eleftherios, Patisia, Rizoupoli, Probona). Its population according to 2011 census is 98,665 (compared to 2001: 105,539).
3. 6th Municipal Community (D6): It includes the central districts (America Square, Attica Square, Kipseli, Nea Kipseli, Ano Kipseli). Its population according to 2011 census list is 130,582 (compared to 2001: 162,366)

In addition, HCMHR serves the Municipality of Galatsi, but the same geographical area is also served by the Community Center for Mental Health of Children and Adolescents of the General Hospital Sotiria (located in Attica Square). This means that according to the 2011 census, a total of 314,876 residents are served by just one center in population level. In 2019, the HCMHR staff consists of 13 persons in total (two administrative clerks, three pediatric psychiatrists, five psychologists, two social workers and one speech therapist), which is considered adequate in accordance with the current operating framework.

The services provided by the HCMHR are particularly important as they are offered to children and adolescents with mental and developmental disorders and psychosocial functioning difficulties (such as dyslexia, autism spectrum disorders, anxiety or behavioral problems). These services include (a) diagnostic evaluation (and possibly evaluation related to cognitive and developmental skills) that could be communicated to school or other relevant stakeholders (e.g. cases of learning disabilities or when there are families applying for an insurance fund, when there are indications of special treatment plans) and (b) treatment.

According to the latest statistics, and specifically for the period from October 10, 2018 to October 10, 2019, the center had 445 new incidents covering all ages (0–18 years). Particular issues related to the whole process may arise from subgroups of populations due to different ethnicity and cultural identity (for example working with interpreters in case of referrals concerning refugee children), but in general there is representation from all social groups, so the HCMHR can be seen as a general system at the patient level that interacts with other systems at a population level (e.g. schools).

The usual procedure followed by HCMHR with the introduction of a new case (along with the relevant timeplans) is as follows:

1. Introduction (of a new case) stage: families (in some cases even children or adolescents on their own) contact and arrange the booking of an appointment to meet with a specialist at HCMHR (usually there is a duration of one to three weeks until the meeting).
2. Assessment stage: At this stage, learning and developmental difficulties, anxiety and behavioral problems among others are assessed. It is also decided whether the child or adolescent will proceed with a treatment plan. There is a two to five percent (2–5%) dropout rate, where the child may not continue or is referred to an external service. At this stage, other specialists may be included for additional sessions.
3. Treatment stage: depending on each case, psychotherapy, counseling, or speech therapy sessions may be held. These sessions are held on a weekly basis (as far as the pediatric psychiatrists are concerned) and once every 15 days with a counseling expert, for a one year period. There is also the possibility of a supportive medication plan (if deemed necessary). These sessions last 45 min and take place on a weekly basis with an average of 40 sessions per week (this number refers to sessions held by all HCMHR specialists), without taking into account the time needed for the consultation.
4. Re-evaluation stage: at the end of treatment, the condition is reviewed and the case is considered 'closed'. Usually 45–50% of cases have completed their cycle by the end of the year.

An indicative example of a new case at HCMHR could be described as such:

1. Parents concerned about their children communicate by telephone with the HCMHR. An appointment meeting is scheduled where both parents and the child meet with a specialist within one week.
2. During the meeting the specialist has a discussion with the parents and the child and concludes with a diagnosis of whether or not a therapeutic regimen may be available (depending on the conclusion reached by the specialist after the first meeting, the regimen could be applied with him/her or with more specialists). In some occasions, the case may also be referred to an external service.
3. After their diagnosis, the child/adolescent and their parents are informed within one to two weeks of the findings in respective sessions. If there is a positive decision from both parties (parents and child), they proceed in a therapeutic plan, depending on the case. Psychotherapy, counseling and/or speech therapy sessions can be held. The sessions that take place, depending on the incident, last for one year and are held on a weekly basis (pediatric psychiatrist) and on a biweekly basis with counseling, possibly followed by a medication plan (if necessary). The sessions last for 45 min.
4. At the end of treatment, the condition is reassessed (related certification is provided upon request from parents) and the case is considered 'closed'.

An event of particular interest is when there are too many new incoming cases preventing the HCMHR from being able to cope with their number. In this case, the HCMHR system "survives" through internal response mechanisms, limiting the therapeutic hours (therapeutic framework) it offers. The uncovered population is served by external services and private entities, however this is particularly important because, as already mentioned, HCMHR tries to serve mainly families based on low financial context, so there is a high likelihood that these families will not receive any treatment because of its accompanying cost. This adaptability feature shares common characteristics with living systems, which “adapt to a continually changing environment and to handle stress from both within and without” [2]. This serves as an inspiration for the transformation of the HCMHR into a general system.

### Existing status of mental health services

Recent literature indicates a lack of quality, efficiency and effectiveness in mental health care services [3]. While mental health is progressively acknowledged as a global health and socioeconomic development priority, several aspects including the social, cultural and medical criteria of the population have not been taken into consideration, as this was investigated in the case of the Japanese people [4] and the South African populations [5]. As far as children are concerned, in order for mental healthcare services to be effective, they should be brought closer to the community with elements of care and efficient use of interprofessional teams [6, 7]. Moreover, previously unexplored evidence e.g. ambient temperature [8] and urban-related problems affecting families, such as housing affordability [9] should also be taken into consideration upon providing mental healthcare services.

This continuously changing field of mental health research demands a critical examination and investigation of different strategies and interventions. It is already known that a multidisciplinary approach [10–12] and an evidence-based modelling [13] of care are needed at the onset of mental health problems, in order to compare and understand the underlying mechanisms of the mental well-being. Therefore, in order to improve the quality and effectiveness of the provided mental health services, an assessment of the existing services’ quality is necessary, as well as “measuring and quantifying it in such a way so that comparisons can be made feasible over time at local, state, and transnational levels” [14]. General Systems Theory framework provides a strong ground for the decision makers to implement mechanisms of depicting and simulating characteristics of systems under investigation from different levels of approach.

### General systems theory and biopsychosocial model in mental health

The foundation of General Systems Theory (GST) as this was introduced by Ludwig von Bertalanffy [15] have been recently brought into spotlight by Tramonti et al. [16] in an effort to re-examine the understanding of mental processes and psychological functioning along with the conceptual foundations for a variety of psychological constructs. The most commonly used derivative of GST is the Biopsychosocial (BPS) model, which is commonly mentioned in mental health care [17, 18] along with its criticism [19]. However an actual operating framework based on GST is not actually put into perspective to interested stakeholders, as it was pointed out by Sharma et al.[20], where they assessed the clinical implementation of BPS in temporomandibular and other orofacial pain conditions. Although evidence and information might be collected from across all of the three general BPS domains, these might not be comprehensive with regard to the individual BPS components, especially when cultural aspects and societal expectations are among the most influential factors in mental health. Evidence-based and multi-level assessment related to the criteria, indicators, and methodology for evaluating and improving the quality of mental health services and their related qualitative and quantitative indicators [14] are deemed more than necessary.

## Methodology

Based on the aforementioned concepts, the aim is to create a comprehensive operational framework based on GST for better illustrating the suggestive time- and action-related field of a mental health treatment and research center. It is suggested that a GST-based mathematical framework will allow a better monitoring and estimation of parameters affecting a mental health system from a micro-, meso- and macro-point of view at any given state of time.

This is necessary in order to identify the relevant actions and mechanisms within the investigated HCMHR system upon facing a variety of cases with children and adolescents facing psychological and developmental problems (e.g. dyslexia, anxiety and behavioural problems). To better support this, we take into consideration the actual operating framework of the HCMHR and convert it into a general system on a patient-level, interacting with a general system of a population-level (e.g. school).

### Calculation of the GST-framework for the HCMHR

**Fig. 2:**
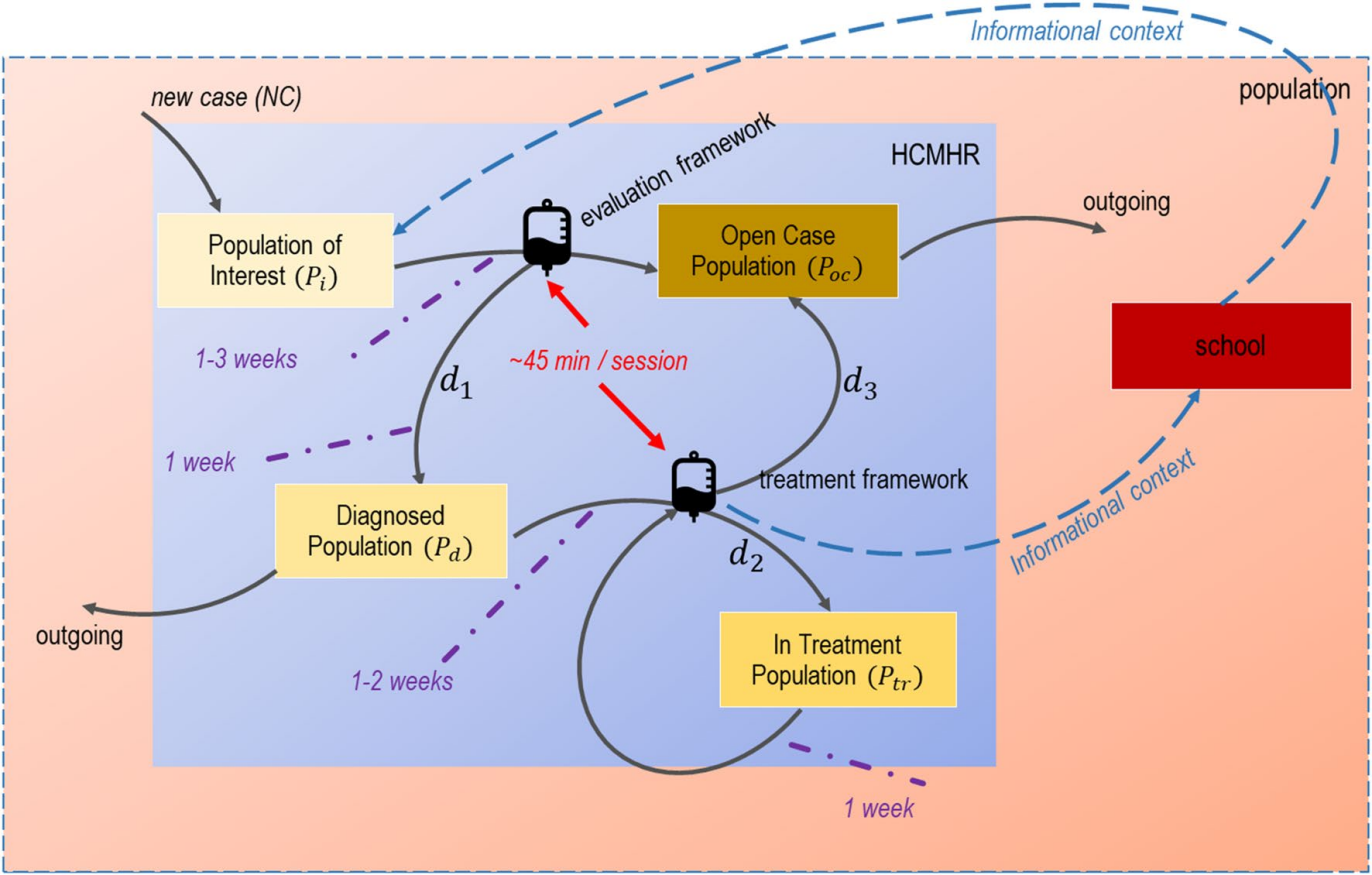
GST operational framework of HCMHR

Based on the foundational framework of GST [15], we first have to define HCMHR’s boundaries. HCMHR is a public organization of private law, therefore its network of mental healthcare units and services comprise its boundaries. We then have to define the elements and the relations (interactions) of the system, as well as its purpose:

1. Population stocks (rectangular boxes in Figs. 2 and 3) indicate the elements of the system which change over time. These involve the population of interest (incoming cases), the diagnosed population, the treatment population and the ‘open cases’ population (i.e. cases that have been already evaluated and/or become open).

**Fig. 3:**
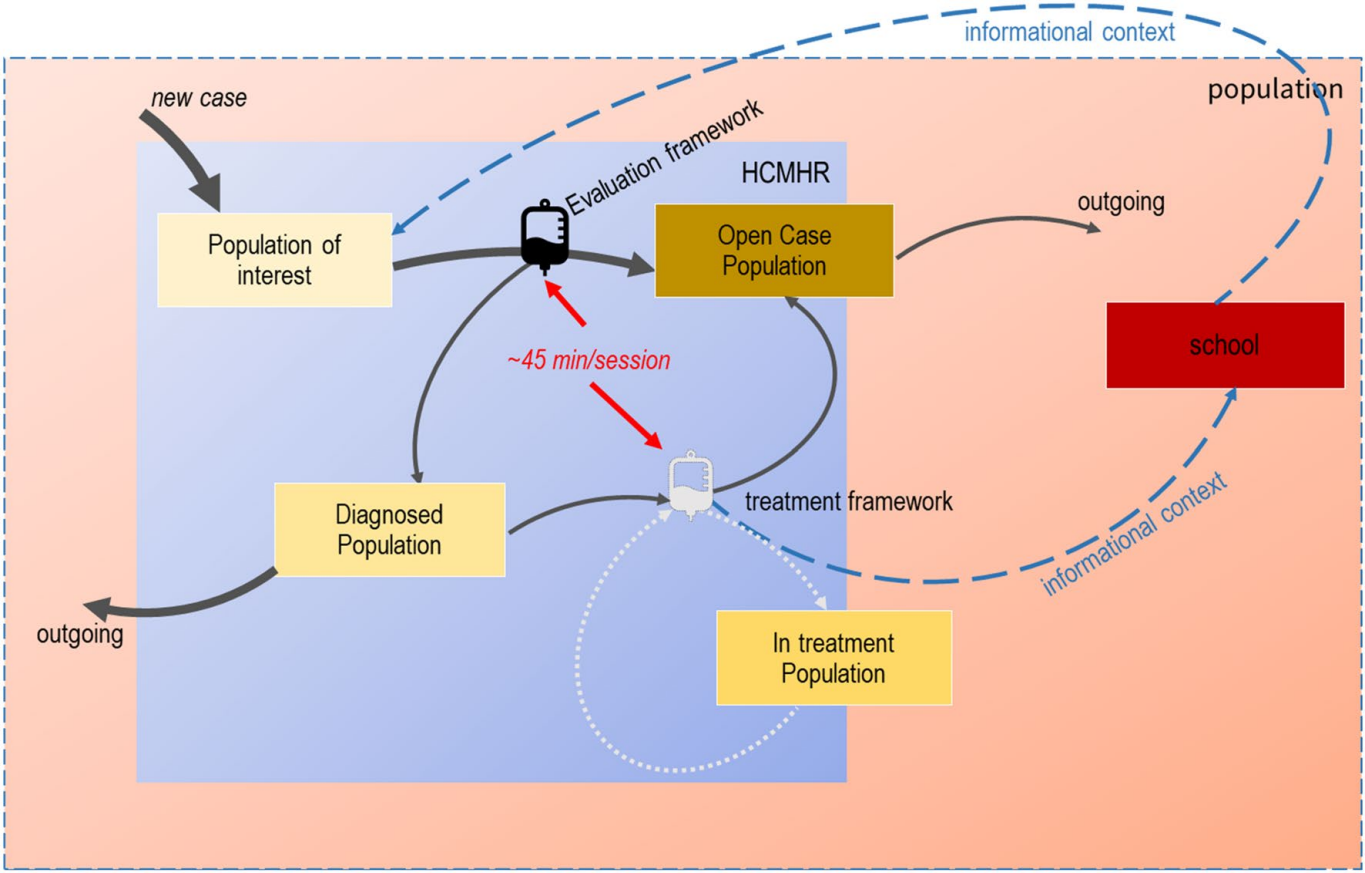
Operational framework of HCMHR under-stress
2. Information- and evaluation-based relationships (flows) (signified by a ‘drip-counter’ icon in Figs. 2 and 3) include the evaluation and the treatment plan frameworks’ rate.
3. The purpose of the HCHMR is (a) to assess the mental, cognitive and behavioural health of the population and (b) to provide a certified assessment (if needed) based on (a).

The next step is to define the states of the system, as well their related time framework. There are conditions of stationary state that should be taken into consideration, where no changes happen (i.e. no incoming cases or disestablishment of the organization), however this is a pretty unlikely event given the present situation. The related flow rates are hereby depicted:

- New case rate: new cases + 50% previous cases
- Evaluation rate: 40 sessions per week
- Diagnostic and therapeutic framework rate: 25–30% of cases per week
- Re-evaluation rate: ~ 45–50% number of re-reviews per year

Given this information, we can construct the following tables (Table 1 and Table 2) of equations depicting the state of the populations in HCMHR at a relevant timeframe (time in our case is considered in respect to the information provided, therefore we define it in our case in weeks):

**Table 1:** State equations of the HCMHR general system

$${S}_{{y}_{0}}^{{P}_{i}}=\frac{1}{2}{S}_{{y}_{52}}^{{P}_{oc}}$$ (1)
$${S}_{{t}_{n}}^{{P}_{i}}=0.98NC\left({t}_{n}\right)+{S}_{{t}_{n-1}}^{{P}_{i}}$$ (2)
$${S}_{{t}_{n}}^{{P}_{d}}=0.98\left({S}_{{t}_{n-1}}^{{P}_{d}}+0.3{S}_{{t}_{n+{d}_{1}}}^{{P}_{i}}\right)$$ (3)
$${S}_{{t}_{n}}^{{P}_{tr}}={S}_{{t}_{n-1}}^{{P}_{tr}}+0.3{S}_{{t}_{n+{d}_{2}}}^{{P}_{d}}$$(4)
$${S}_{{t}_{n}}^{{P}_{oc}}=0.7{S}_{{t}_{n}}^{{P}_{i}}+\frac{1}{2}{S}_{{t}_{n+{d}_{3}}}^{{P}_{tr}}$$ (5)

**Table 2:** Nomenclature

Variable	Description	Units
${y}_{0}$	Starting of the current timeframe	Week
${y}_{52}$	Ending of the previous timeframe	Week
${t}_{n}, where n=[\mathrm{1,51}]$	Present timeframe	Week(s)
${t}_{n-1}$	time of a previous situation	Week(s)
$NC({t}_{n})$	Number of new cases for a given timeframe	Positive Integer
${P}_{x}, where x=\{i,d,tr,oc\}$	Population of interest, diagnosed, in-treatment and open-case	Positive integer
${S}_{{t}_{n}}^{{P}_{x}}, where x=\{i,d,tr,oc\}$	Status of a population at a timeframe	Positive integer
${d}_{1}=\{\mathrm{2,3},4\}$	Transition timeframe of moving from ${P}_{i}\to {P}_{d}$	Weeks
${d}_{2}=\{\mathrm{1,2}\}$	Transition timeframe of moving from ${P}_{d}\to {P}_{tr}$	Weeks
${d}_{3}\le 52$	Transition timeframe of moving from ${P}_{tr}\to {P}_{oc}$	Weeks

In more detail:

- Eq. (1) shows the starting state of the Population of interest ($${P}_{i})$$ at the starting of a new timeframe $$\left({y}_{0}\right)$$ where it includes the 50% of the previous timeframe’s $$\left({y}_{52}\right)$$ open cases $$\left({P}_{oc}\right)$$
- Eq. (2) shows the state $$\left({S}_{{t}_{n}}^{{P}_{i}}\right)$$ of the Population of Interest ($${P}_{i})$$ at the current timeframe $$\left({t}_{n}\right)$$. This consists of any new cases that might occur in that time $$\left(NC\right)$$, taking also into account a drop-out rate of 2%.
- Eq. (3) shows how the state of the Diagnosed Population $\left({S}_{{t}_{n}}^{{P}_{d}}\right)$ changes over the course of the current timeframe, where it has the 30% of the new cases diagnosed $\left({S}_{{t}_{n+{d}_{1}}}^{{P}_{i}}\right)$ along with the previous weeks’ diagnosed cases $\left({S}_{{t}_{n-1}}^{{P}_{d}}\right)$. A drop-out rate of 2% has also been taken into consideration in this case.
- Eq. (4) shows the state of In-Treatment Population at the current timeframe $$\left({S}_{{t}_{n}}^{{P}_{tr}}\right)$$. The same logic with Eq. (3) is applied here, as 30% of the newly starting In-Treatment Population $$\left({S}_{{t}_{n+{d}_{2}}}^{{P}_{d}}\right)$$ increases previous week’s Diagnosed Population ($${S}_{{t}_{n-1}}^{{P}_{tr}})$$
- Finally, Eq. (5) shows the state of the Open Case Population $\left({S}_{{t}_{n}}^{{P}_{oc}}\right)$. This includes the 70% of the not-diagnosed population $\left({S}_{{t}_{n}}^{{P}_{i}}\right)$ and 50% of those who have completed their treatment plan $\left({S}_{{t}_{n+{d}_{3}}}^{{P}_{tr}}\right)$.

These five equations provide a raw logico-mathematical form of the HCMHR as a general system. The solutions to the system at a given year, provide the stability or stationary state that allows HCMHR to continue its existence with increasing time.

## Results

The diagrammatic representation of the above points is depicted in Fig. 2. The blue rectangle represents the patient-level HCMHR system, while the red one shows the population system (in our case the three districts). An indicative population-level system is also depicted (school). Arrows indicate movement from one state to another (that means when an individual patient moves from one population to another inside the same system), along with their respective times (purple dotted lines). This is also in accordance with the indicative example presented in Sect. 2.1.

Figure 3 shows the under-stress functionality framework of HCMHR, when it has too many incoming cases. Arrows weight indicate the amount of movement in each stage/state. In that case, the treatment framework is reduced (faint-lighted colour) as HCMHR does not provide a treatment solution to all diagnosed cases. Therefore, a reduced amount of the in-treatment population stays within the boundaries of the HCMHR system, while the remaining one relies in external, private services. Informational context may be communicated upon parent approval (blue dotted arrows).

The graphical representation of these situations provides a user-friendly process diagram which is easily comprehensible and adjustable, based both on time- and operation-scale.

## Discussion

Accessible and evidence-based approaches on a multi-level perspective need to be integrated in mental health prevention, while addressing the risks in early interventions. Open systems are by de-facto more difficult to be established and described because of the lack of absolute criteria defining their exact states. The difficulties are not only in the complexity of phenomena but in the definition of entities under consideration.

That is to say that our approach has its limitations. We might not have included all possible parameters affecting the states of each population and internal state mechanisms have not been fully analysed. Prospective and longitudinal studies of HCMHR may provide additional insight into the quantitative and qualitative action mechanisms with respect to both patients and center’s staff. Moreover, due to General Data Protection Regulation (GDPR) restrictions, information related to sensitive populations as the ones described here, could not be presented in full detail.

However, it is our intention to have such approaches and tools used within the HCMHR system, allowing it to tackle existing limitations and further enhance a multivariate analysis of its services by applying a GST framework. This includes a further integration of (big) data analytics and simulation models within mental health services centers, to provide evidence-based approaches on a micro- and meso-level. It is also suggested to take into account macro-level interactions (e.g. legislative and financial frameworks) with direct reference to the environment, as this is defined by any observer to the system.

Practical frameworks as the one presented here can be used to guide mental health investigators towards integrating clinical data with culture, social and other types of patient-, population- and nation-related data. Timely identification of risks may build capacity and cost-effective solutions for healthcare centers’ operations, and at the same time make significant contributions into mental health research field.

## Conclusions

In conclusion, the results of this study have provided a detailed approach of a GST framework in a mental healthcare center. Although GST might be a thought-provoking approach requiring different tools from several sciences (including biological, social and mathematical ones), it involves the explicit use of theory and of models, and above all of generalization. This is the most challenging and interesting task in any science or field of knowledge. Nonetheless, the overall adaptation of such approaches is encouraging. The case study presented in our paper stressed out the population aspects of such systems in time. Although there is a number of limitations to certain aspects, the majority of the specific system entities were satisfactorily described within the new framework guidelines. As such, we would suggest implementing new principles of explanation through the diffusion of evidence-based tools and data driven methods. It would be advantageous to include additional states’ simulation or processes as well events related to mental health evaluation and treatment frameworks, to increase overall awareness and inclusion of the meso- and macro-level perspectives.

The conclusion to be drawn from the above is that all mental healthcare systems should focus and compete for the time and energy of the individual patients, especially when they belong to sensitive subgroups, such as children and adolescents; the maintenance of their way of life depends on an equilibrium among systems. Culture, social and personal change comes about through minor variations in one or more systems which grow, displace or reinforce others and reach equilibrium on a different plane. The strategy of the GST approach is therefore to incorporate each system and study it as a time-state vector. The ultimate goal is the real-time reconstruction of the entire pattern of systems interactions to describe the processes by which changes in one field of activity (subsystem) sometimes act to promote changes in other fields (subsystems) and in turn act on the original subsystem itself in a multi-system environment.

## Data Availability

Data sharing is not applicable to this article as no datasets were generated or analysed during the current study.

## Footnotes

1 https://www.who.int/mental_health/maternal-child/child_adolescent/en/
